# Outbreak of *Vibrio parahaemolyticus* Sequence Type 120, Peru, 2009

**DOI:** 10.3201/eid2207.151896

**Published:** 2016-07

**Authors:** Narjol Gonzalez-Escalona, Ronnie G. Gavilan, Magaly Toro, Maria L. Zamudio, Jaime Martinez-Urtaza

**Affiliations:** US Food and Drug Administration Center for Food Safety and Applied Nutrition, College Park, Maryland, USA (N. Gonzalez-Escalona);; Instituto Nacional de Salud, Lima, Peru (R.G. Gavilan, M.L. Zamudio);; Universidad de Chile, Santiago, Chile (M. Toro); University of Bath Milner Centre for Evolution, Bath, UK (J. Martinez-Urtaza)

**Keywords:** Vibrio parahaemolyticus, whole-genome MLST, multilocus sequence typing, clonal complex, next-generation sequencing, sequence type, Peru, El Niño, outbreak, bacteria, bacterial infection, sequence type

## Abstract

In 2009, an outbreak of *Vibrio parahaemolyticus* occurred in Piura, Cajamarca, Lambayeque, and Lima, Peru. Whole-genome sequencing of clinical and environmental samples from the outbreak revealed a new *V. parahaemolyticus* clone. All the isolates identified belonged to a single clonal complex described exclusively in Asia before its emergence in Peru.

*Vibrio parahaemolyticus* is a marine bacterium considered to be one of the major causes of bacterial foodborne outbreaks. Infections caused by *V. parahaemolyticus* have shown a steady expansion in recent years, with a growing number of cases detected worldwide (*1**–**7*).

The epidemiology of *V. parahaemolyticus* infections in Peru has traditionally been dominated by a characteristic pattern of an increase number of cases during the summer months, corresponding to higher coastal water temperatures (*8*). This seasonality in the epidemic dynamics of *V. parahaemolyticus* infections was only altered during the emergence of cases associated with 2 major outbreaks of illnesses reported in the country, which were caused by the arrival of novel genetic variants coming from Asia (*9**,**10*). *V. parahaemolyticus* infections in Peru had been predominantly associated with the O4:K8 serotype and sequence type (ST) 88 until 1995 (*11*), when a novel genetic variant of O4:K8 emerged in the country. Infections caused by this novel variant (ST-189a) quickly spread throughout the country, replacing those caused by the ST-88 variant (*10*). ST-189a was replaced in 1997 as the dominant ST by the arrival of a new variant, the pandemic clone ST-3, which also originated in Asia (*8*,*12*). Infections were mostly associated with the pandemic clone throughout 1997 and 1998 and then with a less clear pattern of dominance afterwards because of the presence of multiple serotypes.

## The Study

A new and large *V. parahaemolyticus* outbreak was detected in Peru during the austral summer of 2009. During February–March 2009, a total of 30 isolates were obtained from clinical samples of patients with symptoms of gastroenteritis. Initially illnesses were reported only in the northern cities of Peru (Cajamarca, Chiclayo, and Piura), but subsequently the outbreak extended to Lima.

Thirty *V. parahaemolyticus* strains isolated from this outbreak were initially investigated for the presence of virulence-related genes, serotyped, and subtyped by using pulse-field gel electrophoresis (PFGE). All strains belonged to serotype O3:K59, a serotype not previously identified in Peru; moreover, all were *tdh*-positive, *trh*-negative, and carried genes for the α variant of the type-3 secretion system 2 (T3SS2α). PFGE analysis showed that all the clinical strains shared an indistinguishable PFGE pattern (Technical Appendix Figure 1).

Environmental strains of *V. parahaemolyticus* isolated from shellfish collected at the central market in Lima over the course of the outbreak were also investigated. These strains (n = 4) were *tdh*-positive, *trh*-negative, T3SS2α-positive, and indistinguishable by PFGE analysis from the outbreak strains.

The genomes of 20 of those strains (18 clinical and 2 environmental) were sequenced by MiSeq (Illumina, San Diego, CA, USA) with 500 (2 × 250) cycles, 2× pair-end library with a minimum coverage of 40–120×; testing was carried out at the US Food and Drug Administration’s Center for Food Safety and Nutrition (College Park, MD, USA). Libraries were prepared with the Nextera XT DNA sample preparation kit (Illumina), according to the manufacturer’s instructions. Whole-genome sequence contigs for each strain were de novo assembled by using CLC Genomics Workbench version 7.5.1 (QIAGEN, Valencia, CA, USA).

In silico multilocus sequence typing (MLST) by eBURST (*13*) identified all strains as belonging to a single sequence type profile, ST-120, which is the ancestral founder of clonal complex (CC) 120 (Technical Appendix Figure 2). All strains deposited in the *V. parahaemolyticus* MLST database belonging to CC120 originated from China. Whole-genome MLST analysis (wgMLST) using Ridom SeqSphere+ version 3.0.0 (http://www.ridom.de/seqsphere) identified 4,265 genes shared among all ST-120 strains from Peru. The genome of strain RIMD 2210633 (*14*) was used as reference. Ridom SeqSphere+ does a gene-by-gene mapping of the shotgun genomes against the reference genome, identifies the core genes present in all genomes, identifies variants at sequence level (single-nucleotide polymorphisms [SNPs]), and assigns alleles to each unique individual gene sequence. SNPs identified in each allele for each locus were extracted and saved into a SNP matrix to be used for further analysis. Then, Nei’s DNA distance method (*15*) was used for calculating the genetic distance matrix by taking the number of same/different alleles scored for each loci in each genome. In some cases, values are not found in certain loci because that gene was either missing or truncated because of its position at either end of the de novo assembled contigs. With these genetic distances, we then built either a neighbor-joining tree or minimum-spanning tree. Among those 4,265 core genes, only 20 were different from the rest. A minimum-spanning tree of these strains showed genetic uniformity among all the outbreak strains, grouping all genomes within a single complex with a central group of 6 strains (Figure). These 6 strains were indistinguishable, and the remaining strains showed minor differences ranging from 1 to 3 alleles and from 1 to 5 SNPs (Technical Appendix Figure 3). Furthermore, environmental strains showed identical allelic profiles and sequences to the outbreak strains, which represent evidence supporting the domestic source of the seafood originating the infections. The shellfish predominantly comprised bivalve mollusk species collected from warm areas of the north of the country where the outbreak originated and that are shipped daily to the central market in Lima.

**Figure:**
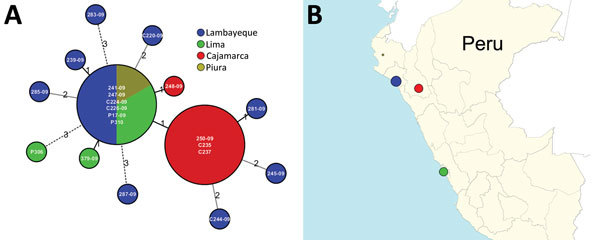
*V. parahaemolyticus* outbreaks in Peru, 2009. A) Minimum spanning tree showing the loci differences among Peruvian sequence type (ST) 120 strains. Ridom SeqSphere+ version 3.0.0 (http://www.ridom.de/seqsphere) identified 4,265 loci shared among all ST-120 *V. parahaemolyticus* strains. The numbers above the connected lines are loci differences. The lines are not to scale. B) Geographic locations of these ST-120 strains in Peru.

A wgMLST analysis of the outbreak isolates with 236 *V. parahaemolyticus* genomes available in GenBank grouped the ST-120 isolates from Peru in a single cluster that exclusively included isolates from China (Technical Appendix Figure 4). This finding constitutes additional support to the findings observed with the use of the available MLST data (Technical Appendix Figure 2), which show genetically similar strains in very distant locations. By wgMLST analysis, ST-120 isolates from Peru differed from 2 isolates isolated in China in 1992 (S016) and 1993 (S018) by 48 and 259 alleles, respectively. The fact that these 2 strains were isolated during the 1990s might explain why they are so different from the Peru ST-120 strains. Expanding the analysis to other genomes of ST-120 recently isolated from China or Southeast Asia might identify more closely related strains.

## Conclusions

Taken together, our findings reveal another example of the emergence of an Asian variant of *V. parahaemolyticus* in Peru associated with seafood consumption. The arrival of ST-120 strains in Peru represents a third instance of an introduction of Asian populations of pathogenic *V. parahaemolyticus* to the Pacific coasts of South America, and, together with the arrival of strains of the seventh pandemic of cholera in 1991, substantiates the existence of recurrent flux of pathogenic *Vibrio* populations between both sides of the Pacific Ocean. Asian and Peruvian coasts are intermittently interconnected through the movement of water displaced by El Niño episodes. These 4 introduction events of pathogenic *Vibrio* strains in Peru occurred just before the arrival of tropical El Niño waters to the Peruvian coasts, which suggests that the introduction of foreign populations of *Vibrio* could be mediated by El Niño events, as previously suggested (*8*).

In conclusion, this study stresses the importance of the application of genomic epidemiology for the routine investigation of outbreaks and surveillance as an efficient and high-resolution tool for tracing the dissemination of pathogens and diseases on a global scale. This latter information is critical to detect the emergence of novel genetic variants, understand the colonization history of pathogens, and assess potential sources and scenarios contributing to the emergence of disease.

Technical Appendix Representative *Not*I restriction patterns and clustering analysis of *Vibrio parahaemolyticus* strains, eBURST analysis showing clonal complex 120, minimum-spanning tree of the Peruvian strains showing single-nucleotide polymorphisms, and neighbor-joining tree showing the high diversity of *V. parahaemolyticus* strains isolated around the world.
