# Supplementary material for: Outbreak of Vibrio parahaemolyticus Sequence Type 120, Peru, 2009
Source: Emerg Infect Dis. 2016 Jul;22(7):1235–7. doi: 10.3201/eid2207.151896 (PMC4918183; doi:10.3201/eid2207.151896)
Supplement: Technical Appendix — Representative NotI restriction patterns and clustering analysis of Vibrio parahaemolyticus strains, eBURST analysis showing clonal complex 120, minimum-spanning tree of the Peruvian strains showing single-nucleotide polymorphisms, and neighbor-joining tree showing the high diversity of V. parahaemolyticus strains isolated around the world. [file 15-1896-Techapp-s1.pdf]

Article DOI: http://dx.doi.org/10.3201/eid2207.151896

Outbreak of *Vibrio parahaemolyticus* Sequence Type 120, Peru, 2009

Technical Appendix

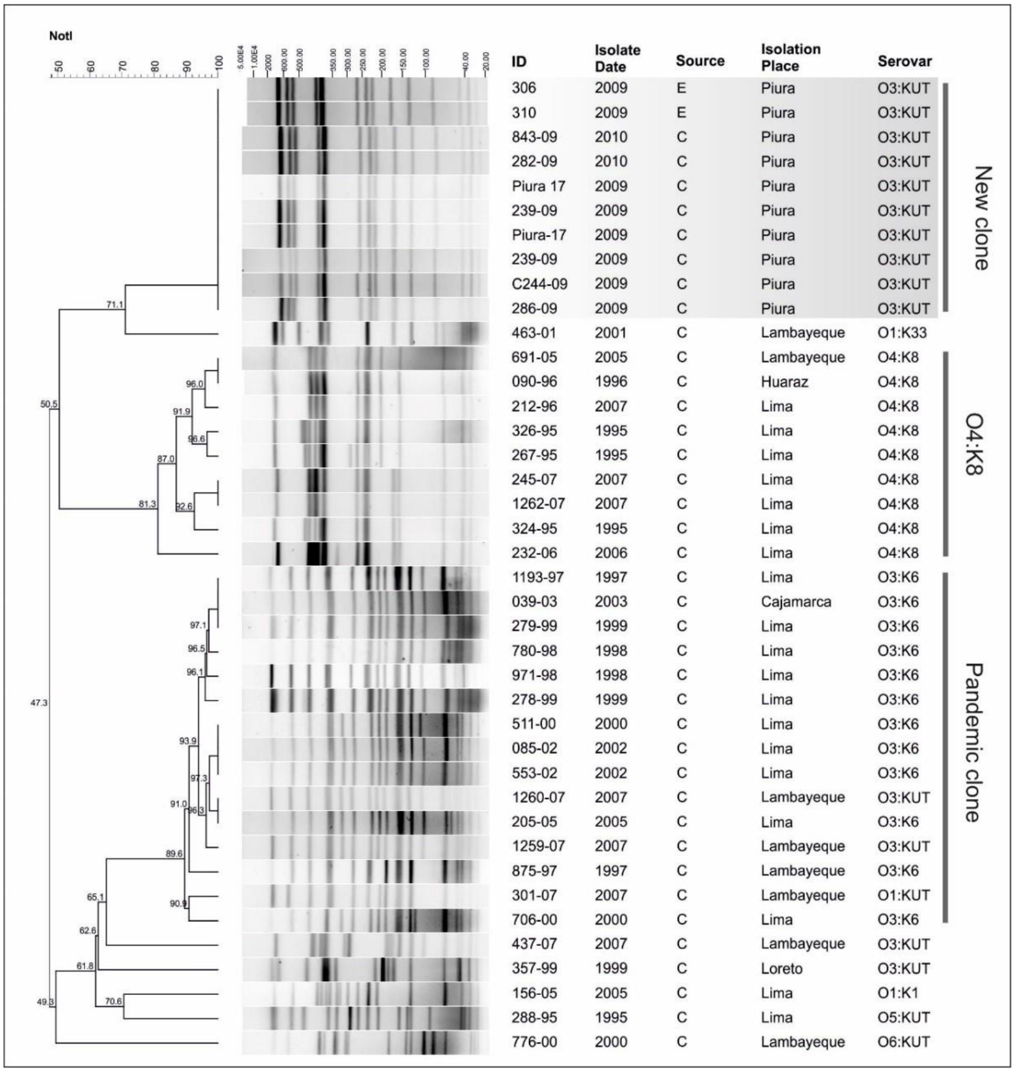

Technical Appendix Figure 1. Representative *Not*I restriction patterns and clustering analysis of *V. parahaemolyticus* strains. Origin, isolation years, pulse-field gel electrophoresis type designations, and O:K serotypes are indicated in the right. Dendrogram generated by using Bionumeric software. Numbers at the dendrogram roots show the similarity value (similarity percentage) among the identified clusters. Numerals at the top of the figure indicate molecular weights (in kbp). Eur, European clone; New, new O3:K6 clone; Old, O3:K6 strains isolated before 1995.

ST	dnaE	gyrB	recA	dtdS	pntA	pyrC	tnaA	clonal complex	Country (# strains Vp MLST)
120	60	108	86	98	18	45	51	120	China (6)
188	60	108	86	99	18	45	51	120	China (1)
333	60	108	31	98	18	45	51	120	China (1)
447	60	108	86	13	18	62	51	120	China (1)
480	60	4	86	98	18	45	51	120	China (2)
663	60	108	86	98	29	45	51	120	-

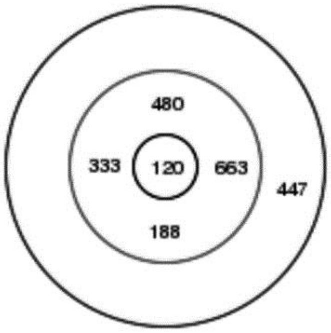

Technical Appendix Figure 2. eBURST analysis showing clonal complex 120. Table shows sequence type members of this clonal complex found in the *Vibrio parahaemolyticus* multilocus sequence typing database and the number of strains with those sequence types.

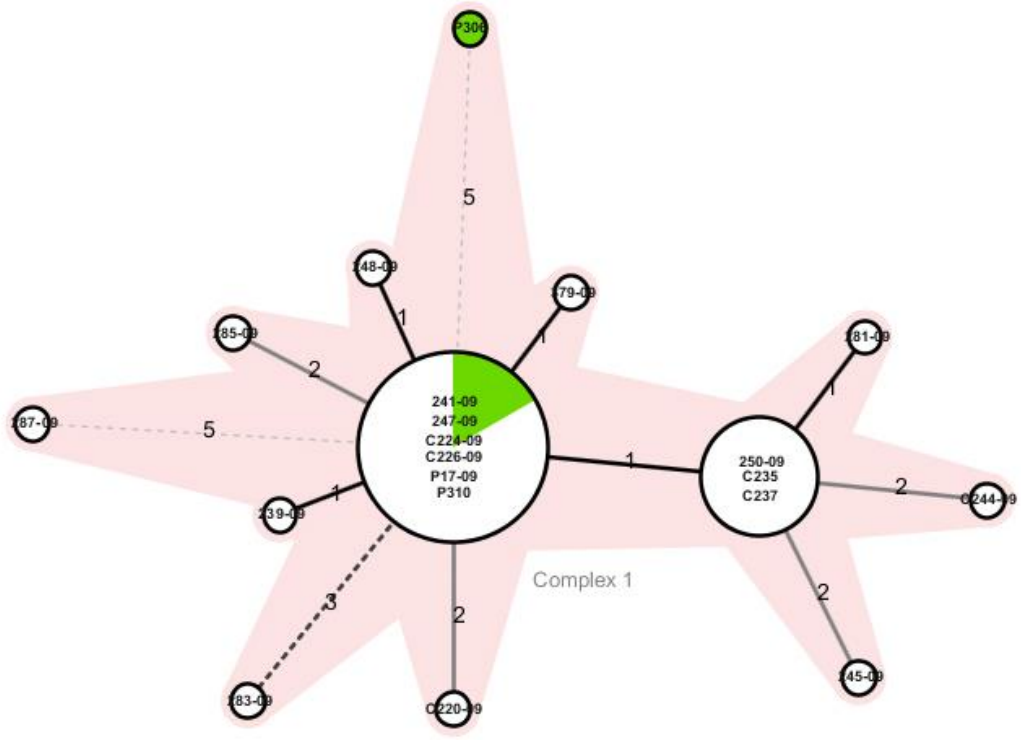

Technical Appendix Figure 3. Minimum spanning tree of the Peruvian strains of *Vibrio parahaemolyticus* showing the amount of single-nucleotide polymorphisms differing among them, the clonality of the strains, and the fact that the seafood's sequence type 120 strains were undistinguishable from clinical sequence type 120 strains. Seafood's isolates shown in green. Numbers above the connected lines are single-nucleotide polymorphisms. Lines are not drawn at scale.

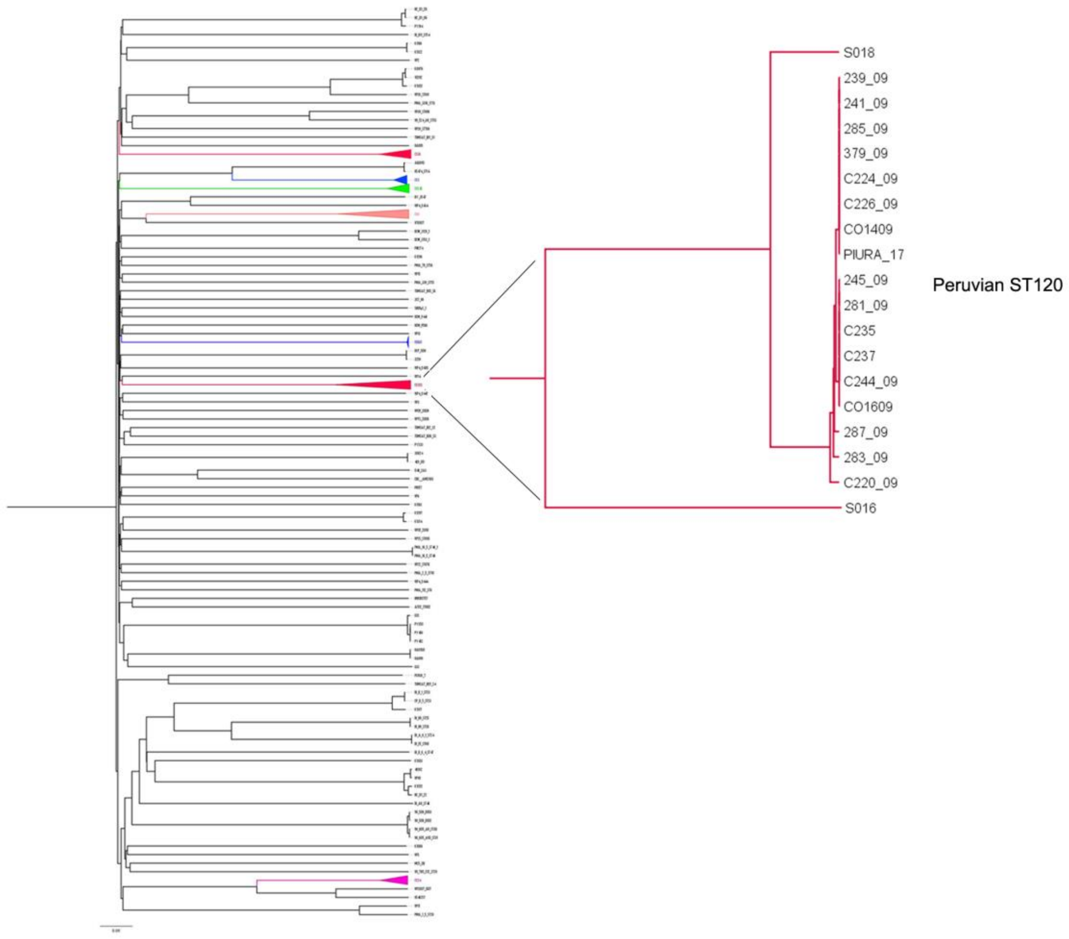

Technical Appendix Figure 4. Neighbor-joining tree showing the high diversity of *Vibrio parahaemolyticus* strains isolated globally and the placement of the Peruvian strains within strains belonging to sequence type 120 from China (1,683 loci were shared among the 236 genomes available at GenBank).
